# A Novel Device to Improve the Tolerability of Intranasal Corticosteroid Sprays for Allergic Rhinitis and Postoperative Chronic Rhinosinusitis: A Crossover Trial

**DOI:** 10.1111/coa.70137

**Published:** 2026-06-27

**Authors:** Sethmi Ranasinghe, Xenia Berger, Wandia Kimita, Kristi Biswas, Raymond Kim, Richard Douglas

**Affiliations:** ^1^ Department of Surgery The University of Auckland Auckland New Zealand; ^2^ Te Whatu Ora, Health New Zealand Auckland New Zealand; ^3^ Department of Otolaryngology‐Head and Neck Surgery Auckland City Hospital Auckland New Zealand

**Keywords:** AR, corticosteroids, CRS, device, FESS, intranasal, postoperative, SNOT‐22

## Abstract

**Background:**

Allergic rhinitis (AR) and chronic rhinosinusitis (CRS) are commonly treated with intranasal corticosteroid sprays. Despite their efficacy, unpleasant sensations associated with their application, including post‐nasal drip and nasal irritation, are often cited as reasons for poor compliance. Optimising the intranasal application experience is key to improving long‐term adherence. We developed a nasal adaptor for metered‐dose inhalers (MDIs) to reduce the unpleasantness of nasal administration, potentially improving patient compliance.

**Methods:**

Two crossover trials were conducted in AR and postoperative CRS participants (*n* = 16 per group). Participants used a standard fluticasone nasal spray (50 μg) and a fluticasone MDI (125 μg) with the adaptor for 3 weeks each, separated by a 1‐week washout period. Device order was alternated, and doses were adjusted to ensure an approximately equal fluticasone dose. Device preference was determined using an adjusted Clinical Trial Patient Preference Questionnaire, and symptom improvement was assessed using Total Nasal Symptom Score and Sino‐nasal Outcome Test‐22 questionnaires.

**Results:**

Symptom improvement was comparable between the devices. However, in both groups, 11/16 (69%) participants preferred the MDI with the adaptor over the standard nasal spray, citing fewer unpleasant sensations. Satisfaction score improvement was significantly greater with the novel device in the CRS cohort. Participants reported a higher likelihood of compliance with the novel device, suggesting improved adherence.

**Conclusions:**

An MDI with the adaptor was preferred over a standard nasal spray among AR and postoperative CRS participants. The enhanced tolerability may promote improved compliance, and consequently, more effective long‐term symptom control.

## Key Points

- A key barrier to the use of intranasal corticosteroid sprays in the management of AR and CRS is the unpleasant sensory experience associated with conventional nasal spray devices, which often leads to poor adherence and suboptimal symptom control.
- To address this, we developed a novel nasal adaptor compatible with a metered‐dose inhaler, designed to improve user comfort, satisfaction, and treatment compliance.
- Crossover studies were conducted in participants with AR and CRS to compare the novel device used with an MDI to a standard nasal spray.
- The majority of participants with AR and CRS preferred the novel device application, primarily due to reduced sensory side effects and improved ease of use.
- Improved satisfaction and adherence associated with the novel device application may translate into more effective long‐term symptom control in AR and CRS.

## 1 Introduction

Allergic rhinitis (AR) and chronic rhinosinusitis (CRS) are highly prevalent inflammatory conditions of the upper airway that significantly impair quality of life and impose a substantial burden on healthcare systems [1, 2]. Despite etiological differences, both are characterised by persistent mucosal inflammation and often require long‐term anti‐inflammatory treatment [1, 2]. Intranasal corticosteroid sprays are safe and effective treatments for AR and CRS [3, 4, 5]. Meta‐analyses demonstrate greater symptom relief with these sprays compared with oral antihistamines for AR symptom management [6]. In CRS patients unresponsive to medical therapy, functional endoscopic sinus surgery (FESS) is indicated to restore ventilation, mucociliary clearance and enhance mucosal drug delivery [7]. Long‐term intranasal corticosteroid treatment has been shown to reduce the rate of recurrence [8].

A key barrier to successful management of AR and post‐FESS CRS is poor adherence to intranasal corticosteroid sprays. Despite their proven efficacy, they are associated with variable drug delivery, which compromises treatment and leads to patient dissatisfaction [3]. A systematic review of 31 studies on intranasal corticosteroid adherence in AR demonstrated wide variability in adherence rates, ranging from 28% to 87% [5]. Sensory attributes, including post‐nasal drip, nasal irritation and unpleasant aftertaste, were among the frequently cited reasons for non‐adherence. Gokani et al. evaluated treatment adherence in 94 post‐FESS CRS patients and found that 66% of participants did not follow their prescribed medication timing or dosage [4]. Reported reasons for non‐adherence were lack of symptom improvement (17%), symptom deterioration (17%) and side effects (10%).

The efficacy of nasal sprays is limited by their delivery mechanics [9]. Standard sprays produce large, high‐velocity droplets that deposit effectively on the anterior aspects of the inferior turbinate, but often fail to reach more posterior regions, particularly in surgically altered sinus [9, 10]. This suboptimal delivery may contribute to inadequate symptom relief, which encourages discontinuation. The challenge may lie not in the pharmacological efficacy of corticosteroids, but in their delivery and tolerability. This highlights the need for alternative delivery methods that improve compliance, symptom control and subsequently, overall quality of life.

We designed a novel adaptor that enables a standard metered‐dose inhaler (MDI) to function as a nasal delivery device (Figure S1). Using a post‐FESS sinonasal cavity perspex model, it was found that this device improves drug deposition in the ethmoid, maxillary, sphenoid and frontal sinuses compared with a standard nasal spray [11]. The novel device delivers medication as a fine mist, the particles of which move more slowly than traditional sprays. These characteristics enhance dose dispersion, suggesting potential for improved tolerability, efficacy and compliance.

The present study primarily compared satisfaction, compliance and preference between the novel adaptor and a standard nasal spray in AR and postoperative CRS participants. The secondary aim was to compare symptom control achieved by the two devices.

## 2 Methods

### 2.1 Novel Nasal Adaptor

The adaptor was printed using *Tough 1500 Resin* (Formlabs, MA, USA) on a Form 3 SLA (stereolithography) 3D printer (Formlabs, MA, USA).

### 2.2 Study Design

This study was designed as two single‐centre, prospective, open‐label, crossover trials. Ethics approval was obtained from the Health and Disability Ethics Committee (ethics number 13240), and all participants provided written informed consent.

### 2.3 Recruitment and Eligibility

AR participants were recruited from the community through flyers. Eligibility required a clinical diagnosis of AR as per the ICAR‐Allergic Rhinitis 2023 guidelines [12], and a baseline Total Nasal Symptom Score (TNSS) [13] of ≥ 4 to ensure sufficient symptom severity to allow for assessment of improvement. Participants were recruited between February and June 2024.

Postoperative CRS participants had been treated at Gillies Hospital and were recruited by a research nurse. Participants were eligible if they met the diagnostic criteria for CRS as outlined in the European Position Paper on Rhinosinusitis and Nasal Polyps (EPOS) 2020 [14]. All participants had undergone FESS more than 3 months prior to recruitment (comprehensive or mini‐FESS procedures). Participants were recruited between June 2023 and June 2024.

Participants were required to be aged between 18 and 80 years and have normal lung function (stable and reproducible baseline FEV1 of > 80% of the predicted value, adjusted for height, age and sex according to the Global Lung Initiative equation) [15]. Exclusion criteria included a history of cystic fibrosis, primary ciliary dyskinesia, hypogammaglobulinaemia, allergy to fluticasone or other topical corticosteroids and recent COVID‐19 infection.

### 2.4 Protocol

Participants received: an instruction sheet, 50 μg/dose fluticasone propionate nasal spray (Flixonase, GlaxoSmithKline), 125 μg/dose fluticasone propionate MDI (Flixotide Inhaler, GlaxoSmithKline), the nasal adaptor, relevant symptom questionnaires (TNSS [13] for AR, Sino‐nasal Outcome Test‐22 [SNOT‐22] [16] for CRS), and an adjusted Clinical Trial Patient Preference Questionnaire (CTPPQ) [17].

Participants began with a 1‐week washout period without the use of intranasal sprays. CRS participants were permitted to use standard saline lavages as needed. All participants were asked to discontinue the use of other intranasal corticosteroids or antihistamines for the duration of the study period. Participants then started treatment with one device for 3 weeks, either the nasal spray (three puffs to each nostril daily) or MDI with the novel adaptor (two puffs to each nostril daily), followed by a second 1‐week washout period. The alternative device was then used for another 3‐week period. We previously determined that approximately 60% of the dose administered via the MDI through the nasal adaptor emerged from the aperture (the remainder adhered to the inner surface) [11]. Accordingly, the total dose per nostril administered by the MDI and adaptor was 125 μg × 2 × 0.6 = 150 μg, which is the same as the dose administered by the standard nasal spray (50 μg × 3 = 150 μg). Participants were assigned in an alternating sequence: the first participant received the standard spray, the second with the MDI, the third with the standard spray, and so on. Symptom questionnaires were completed at baseline and after each treatment period. Figure S2 outlines the timing of questionnaires.

### 2.5 Outcomes

The primary study outcome was device preference, assessed with the CTPPQ, a validated tool for evaluating the tolerability of sinonasal treatments [17]. It comprises three domains: satisfaction, compliance and preference.

Satisfaction was scored from 1 to 7 across eight sinonasal side effects and overall satisfaction. Lower scores indicate greater satisfaction (1 = very satisfied, 7 = very dissatisfied) [17]. Compliance was scored from 1 to 4 for each device. A lower score indicates higher tolerability and compliance (1 = very likely to take, 4 = not likely to take) [17]. Preference was based on participant feedback.

The secondary outcome, symptom control, was assessed using validated symptom questionnaires. AR participants completed the TNSS questionnaire, which scores four common AR symptoms on a scale of 0–3, with higher scores indicating more severe symptoms (0 = symptom not evident, 3 = symptom hard to tolerate and interferes with daily activity) [14]. The minimal clinically important difference (MCID) for AR has been previously defined as a reduction of at least 30% in the maximum score of the questionnaire used, corresponding to a TNSS reduction of ≥ 3.6 points to indicate clinically meaningful improvement [14].

Postoperative CRS participants completed the SNOT‐22 questionnaire [16]. The MCID for the total SNOT‐22 score has been previously defined as 8.9 points [18].

### 2.6 Statistical Analysis

A power analysis was performed for the CRS cohort. For 90% power, a significance level of 0.05, and an expected standard deviation of 7 points on the SNOT‐22 questionnaire, a minimum of 15 participants were required to detect a MCID of 8.9 points (the minimum clinically significant change in SNOT‐22 scores) [18]. This requirement was met, with 16 participants included in the final analysis. An equal number of AR participants were recruited.

Continuous variables were presented as median (IQR). TNSS and SNOT‐22 scores were reported as mean (SD). Statistical analyses were conducted using Microsoft Excel and R 4.2.2 software. A *p*‐value < 0.05 was considered statistically significant.

## 3 Results

### 3.1 Participant Disposition and Characteristics

In the AR trial, 25 individuals were screened, 20 of whom met the eligibility criteria. Two participants withdrew due to adverse effects (headaches, nausea), and two participants were lost to follow‐up. Sixteen AR participants completed the trial and were included in the final analysis. In the CRS trial, 16 eligible participants completed the trial and were included in the final analysis.

A sample size of 32 participants was considered appropriate to assess the feasibility and preliminary efficacy of the intervention in this exploratory pilot study. Participant characteristics are summarised in Table S1. Figure S3 outlines the study flow.

### 3.2 Satisfaction

In the AR trial, the median (IQR) overall satisfaction score was 1 (0.5) for the MDI with the novel adaptor and 3 (3) for the standard spray; *p* = 0.06 (Table 1).

**TABLE 1:** Summary of satisfaction scores presented as median (IQR).

	AR			CRS		
	MDI + adaptor	Standard spray	*p*	MDI + adaptor	Standard spray	*p*
Immediate taste of the solution	1 (1.5)	2 (1)	0.19	1 (1)	3 (2)	0.00003
Aftertaste of the solution	1 (1.5)	3 (3)	0.09	1 (1)	3 (2.5)	0.00002
Smell of the solution	1 (2.5)	3 (3)	0.11	1 (1)	2 (2.5)	0.001
Irritation to your nose	1 (1.5)	3 (4)	0.17	1 (0)	3 (3.5)	0.0003
Urge to sneeze	1 (1.5)	3 (3)	0.28	1 (1)	2 (4)	0.0006
Dripping out your nose	1 (1.5)	2 (4)	0.18	1 (0.5)	5 (4.5)	0.0002
Dripping down your throat	1 (1)	2 (2)	0.31	1 (0)	3 (3.5)	0.00003
Moistness of your nose or throat	2 (2)	2 (2)	0.68	1 (0)	3 (3)	0.00009
Overall satisfaction	1 (0.5)	3 (3)	0.06	1 (0.5)	3 (2.5)	0.0001

*Note:* Lower scores indicate greater satisfaction.

In the CRS cohort, the median (IQR) overall satisfaction score was also 1 (0.5) for the novel device, compared with 3 (2.5) for the standard spray; *p* = 0.0001. The median score was 1 (= very satisfied), and the ranges were narrower with the novel device across all side effect domains (Table 1).

**FIGURE 1:**
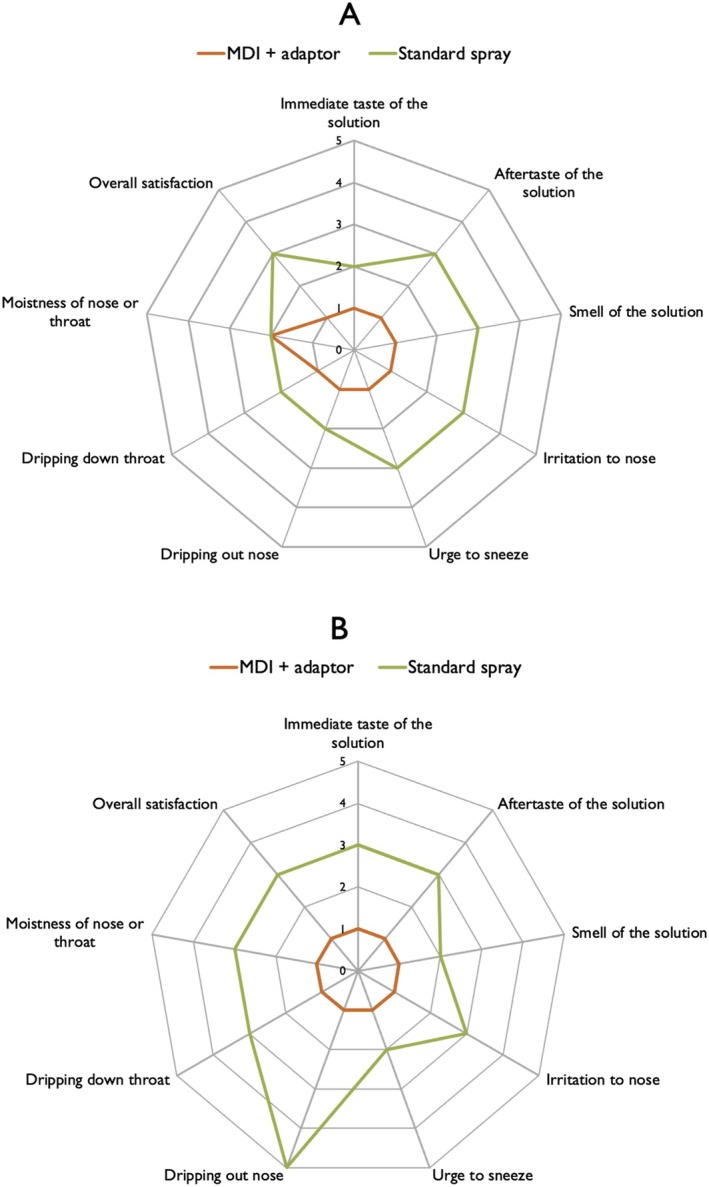
Radar plots displaying median scores for satisfaction across a range of sensory side effects and overall satisfaction, with a lower score indicating greater satisfaction. (A) AR and (B) CRS.

The MDI consistently demonstrated lower scores and narrower ranges than the standard spray. The radar graphs (Figure 1) show tight, symmetrical profiles for the novel device, correlating to higher satisfaction across all domains. The standard spray shows a broader, irregular pattern, suggesting greater variability.

### 3.3 Compliance

CRS participants reported a high likelihood of compliance for the novel device; 13 participants (81%) reported that they would be ‘very likely’ to comply. The median (IQR) compliance score of the MDI with adaptor, 1 (0), was lower than that of the standard spray, which was 2 (1.5); *p* = 0.0005 (Table 2). Notably, four CRS participants (25%) reported that they would not use the standard spray at all, highlighting a considerable risk of non‐compliance.

**TABLE 2:** Summary of compliance scores presented as median (IQR).

AR			CRS		
MDI + adaptor	Standard spray	*p*	MDI + adaptor	Standard spray	*p*
1 (2)	2 (1)	0.64	1 (0)	2 (1.5)	0.0005

*Note:* Lower scores indicate greater likelihood of compliance.

AR participants showed more moderate compliance scores but still demonstrated greater confidence in compliance with the MDI with the adaptor, yielding a median (IQR) compliance score of 1 (2) compared with 2 (1) for the standard nasal spray; *p* = 0.64 (Table 2).

### 3.4 Preference

**FIGURE 2:**
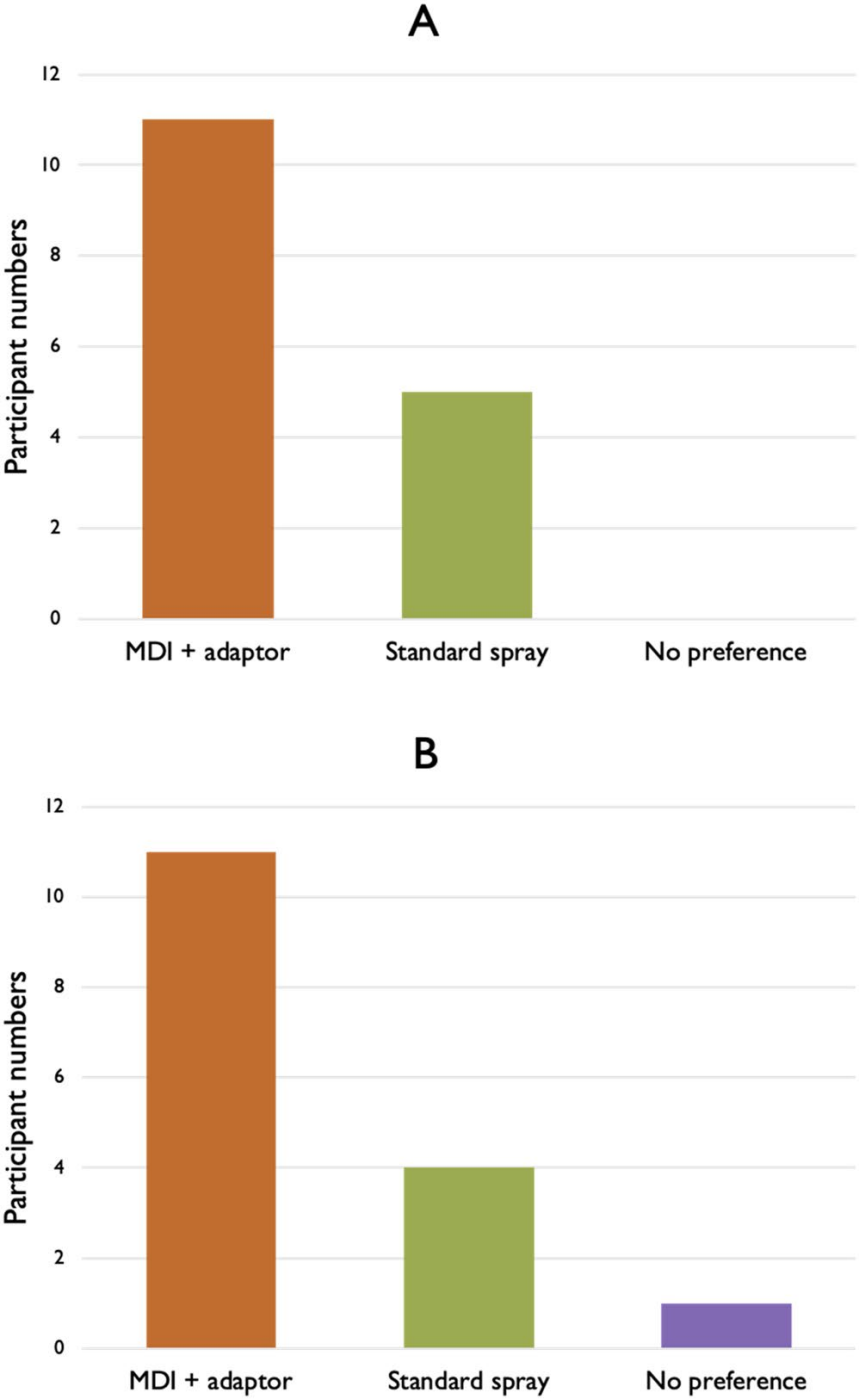
Rates of device preference across the participants, (A) AR and (B) CRS. In both trials, 11 out of 16 participants preferred the MDI with the novel adaptor over the standard nasal spray.

In both trials, 11 out of 16 participants (69%) preferred the MDI over the standard nasal spray (Figure 2). Of the nine participants who preferred the standard spray, most reported that the adaptor felt uncomfortable to use and that they felt accustomed to using a standard spray. Despite this, most of these participants preferred the novel device specifically for managing post‐nasal drip.

### 3.5 Symptom Management: AR Trial

**FIGURE 3:**
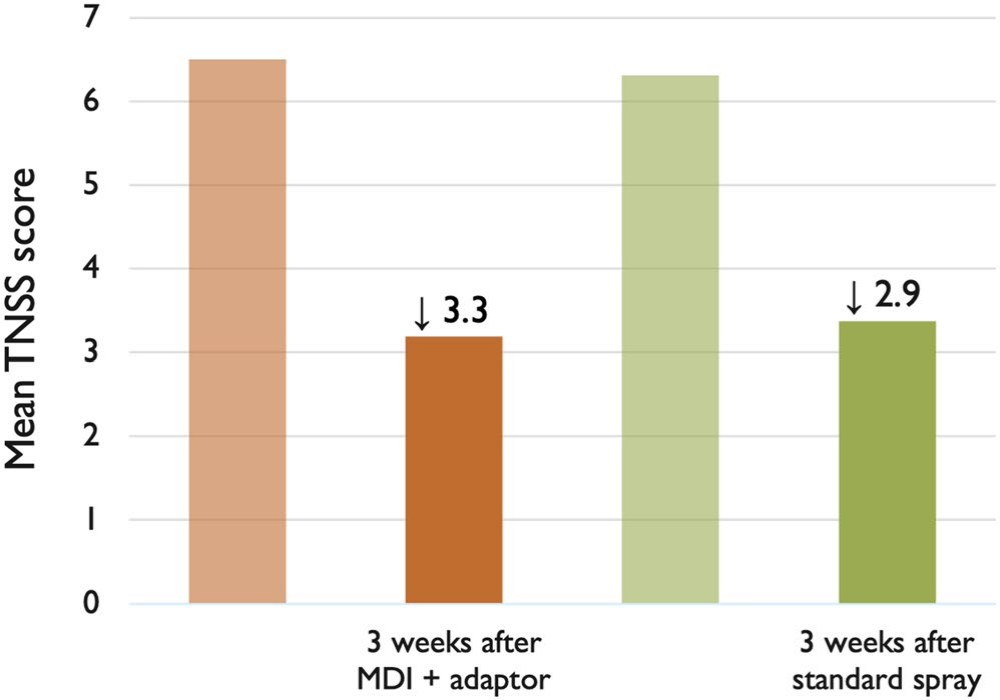
Mean TNSS score reduction in the AR trial after 3 weeks of using each device.

*MDI with adaptor:* The mean (SD) baseline TNSS score was 6.5 (2.6). After 3 weeks of using the MDI, the mean (SD) score decreased to 3.2 (2.3), resulting in a difference of 3.3 points (Figure 3). As this is below the TNSS MCID of 3.6, the results do not meet the threshold for clinical significance.

*Standard nasal spray:* The mean (SD) baseline TNSS score was 6.3 (2.8). After 3 weeks of using the standard spray, the mean (SD) score decreased to 3.4 (3.0), yielding a difference of 2.9 points (Figure 3). This also falls below the MCID.

### 3.6 Symptom Management: CRS Trial

**FIGURE 4:**
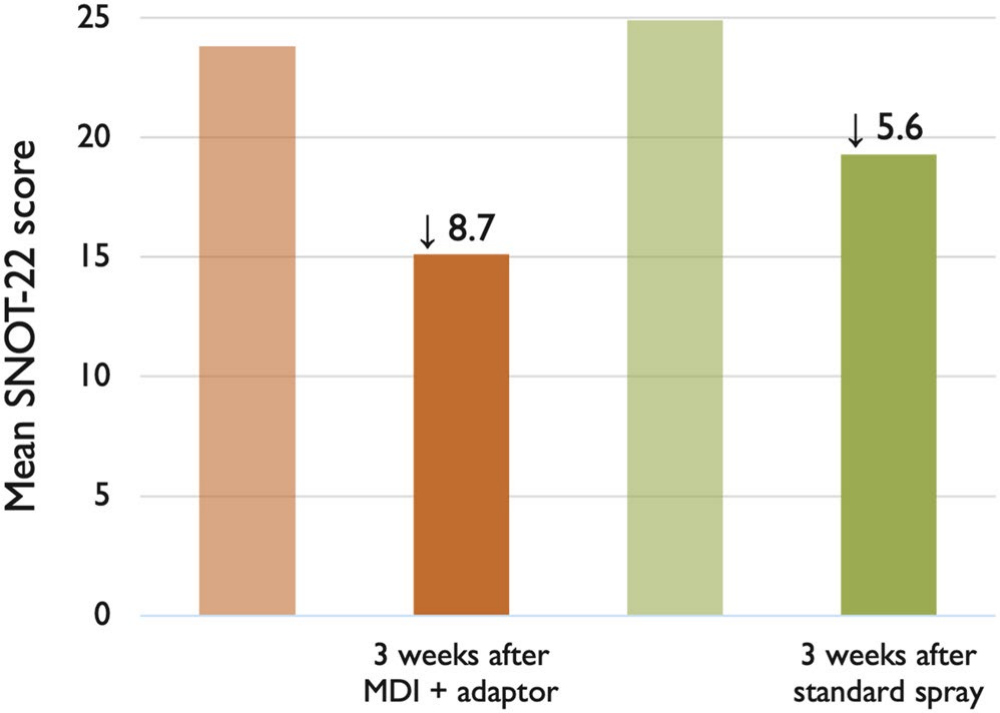
Mean SNOT‐22 score reduction in the CRS trial after 3 weeks of using each device.

*MDI with the novel adaptor:* The mean (SD) baseline SNOT‐22 score was 23.8 (16.7). After 3 weeks of using the MDI, the mean (SD) score decreased to 15.1 (14.6), representing a mean reduction of 8.7 (Figure 4). This score approaches the SNOT‐22 MCID threshold of 8.9.

*Standard nasal spray:* The mean (SD) baseline SNOT‐22 score was 24.9 (18.2). After 3 weeks of using the standard spray, the mean (SD) score decreased to 19.3 (18.1), resulting in a 5.6‐point reduction (Figure 4). This is well below the MCID of 8.9, so it does not correlate with a clinically significant improvement.

## 4 Discussion

Two crossover trials were conducted in AR and postoperative CRS participants to compare device preference and symptom management between an MDI with a novel nasal adaptor and a standard nasal spray. In both trials, a clear majority of participants preferred the MDI. Participants favoured the novel device across all side‐effect domains and reported an increased likelihood of compliance. These findings support the idea that modifying the delivery mechanism may reduce the unfavourable effects of traditional sprays and improve compliance, as it is generally accepted that patients are more likely to adhere to medications with fewer side effects [19].

Satisfaction plays a critical role in health outcomes [20]. Although symptom questionnaire MCID thresholds were not met, participants reported a high likelihood of compliance with the novel device, particularly in the CRS trial. Long‐term adherence is essential in the management of AR and CRS [4, 5], so a delivery method that prioritises the patient experience will likely enhance treatment efficacy.

While TNSS and SNOT‐22 address common symptoms associated with AR and CRS, they are not all‐encompassing. Patients may experience a broader range of issues, including sleep impairment and the psychological burden of chronic symptoms [21]. Mental well‐being is a significant component of patient satisfaction [21], highlighting the importance of not relying solely on symptom scores.

In postoperative CRS patients, high‐volume corticosteroid irrigations tend to be more effective than nasal sprays in severe cases [22]. While our novel device is unlikely to replace these, it provides a cost‐effective option for patients who do not require high‐volume irrigations but dislike using standard sprays.

### 4.1 Limitations

Crossover trials allow participants to act as their own controls, enabling precise comparisons with fewer participants [23]. A limitation of this trial design is the carryover effect, where the first treatment influences outcomes in the second treatment period [23]. To minimise this risk, a 1‐week washout period was implemented between treatment phases. Participants were evenly assigned to each starting device to reduce potential imbalances between devices.

The study enrolled 32 participants, which may limit generalisability. Regardless, it offers valuable insights and a foundation for larger parallel‐group trials. Our study was primarily designed to determine which device was preferred. The relatively small number of patients and short duration of treatment limited the potential of the study to determine which device was more effective. A larger study of longer duration would be required to determine this.

Prior negative experiences with traditional nasal sprays may have influenced participants to favour the novel device. Future studies could enrol nasal spray naïve patients for an unbiased comparison.

Spray delivery varied between the devices, and the higher number of sprays used in each treatment with the traditional device (three vs. two) may have increased side effects, confounding satisfaction outcomes.

The use of self‐reported data introduces potential recall bias [24]. This was minimised through clear instructions and regular reminders. Further studies with larger cohorts could incorporate objective measures, such as endoscopy or Lund–Mckay scoring, to complement the subjective outcomes [25].

## 5 Conclusion

AR and postoperative CRS participants preferred the MDI with the novel adaptor over the standard nasal spray. Symptom improvement was similar for both devices; however, subjects reported greater satisfaction and likelihood of compliance with the novel device. This novel method of intranasal medication delivery may address the commonly experienced sensory side effects associated with standard nasal sprays and improve compliance, thereby improving treatment efficacy for patients with AR and CRS.

## Author Contributions

All authors contributed to the study conception and design. Kristi Biswas and Richard Douglas devised the study idea. Sethmi Ranasinghe, Xenia Berger and Wandia Kimita recruited the participants. Sethmi Ranasinghe, Wandia Kimita and Kristi Biswas completed the data analysis. Sethmi Ranasinghe and Xenia Berger drafted the manuscript, and Wandia Kimita, Kristi Biswas, Raymond Kim and Richard Douglas critically revised the work. All authors read and approved the final manuscript.

## Funding

The authors have nothing to report.

## Ethics Statement

Ethics approval was obtained from the Health and Disability Ethics Committee (#13240), and all participants provided written informed consent.

## Conflicts of Interest

The authors declare no conflicts of interest.

## Supporting information


**Figure S1:** MDI with the novel adaptor.
**Figure S2:** Flowchart demonstrating the timing of questionnaires in both CRS and AR trials.
**Figure S3:** Flowcharts demonstrating the study process in the CRS and AR trials.
**Table S1:** Participant characteristics in the CRS and AR trials.

## Data Availability

The data that support the findings of this study are available from the corresponding author upon reasonable request. The supplier of the 3D‐printed novel nasal adaptor can be contacted at technical@medlink.co.nz.
