# Supplementary material for: A Novel Device to Improve the Tolerability of Intranasal Corticosteroid Sprays for Allergic Rhinitis and Postoperative Chronic Rhinosinusitis: A Crossover Trial
Source: Clin Otolaryngol. 2026 Jun 27;51(5):776–83. doi: 10.1111/coa.70137 (PMC13432547; doi:10.1111/coa.70137)
Supplement: Supplementary file 1 — Figure S1: MDI with the novel adaptor. Figure S2: Flowchart demonstrating the timing of questionnaires in both CRS and AR trials. Figure S3: Flowcharts demonstrating the study process in the CRS and AR trials. Table S1: Participant characteristics in the CRS and AR trials. [file COA-51-776-s001.docx]

# **SUPPLEMENTARY FIGURES**

**

**

**Figure 1** MDI with the novel adaptor. The novel adaptor (left), *Flixotide* inhaler (centre), *Flixotide* inhaler with 3-D printed novel adaptor attached (right).

**Figure 2** Flowchart demonstrating the timing of questionnaires in both CRS and AR trials.
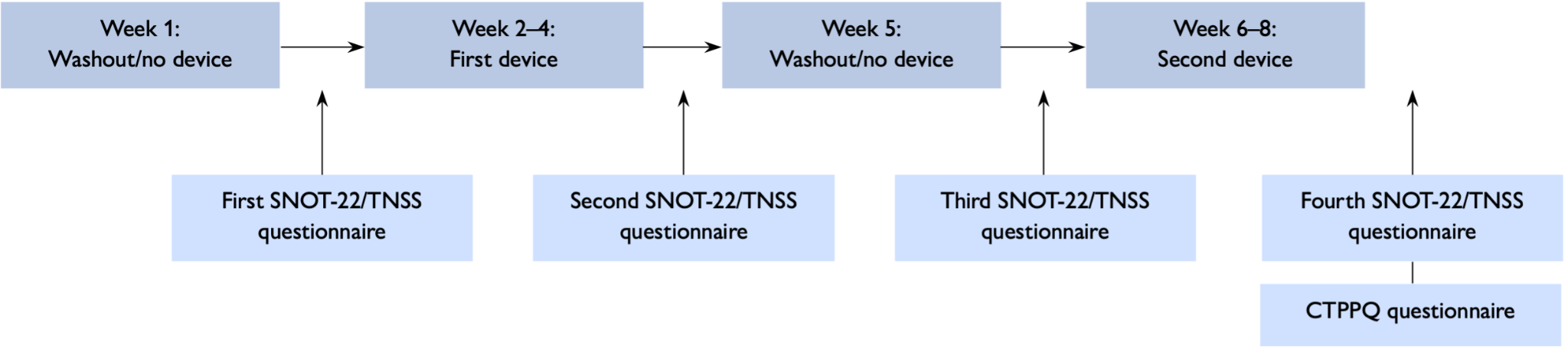


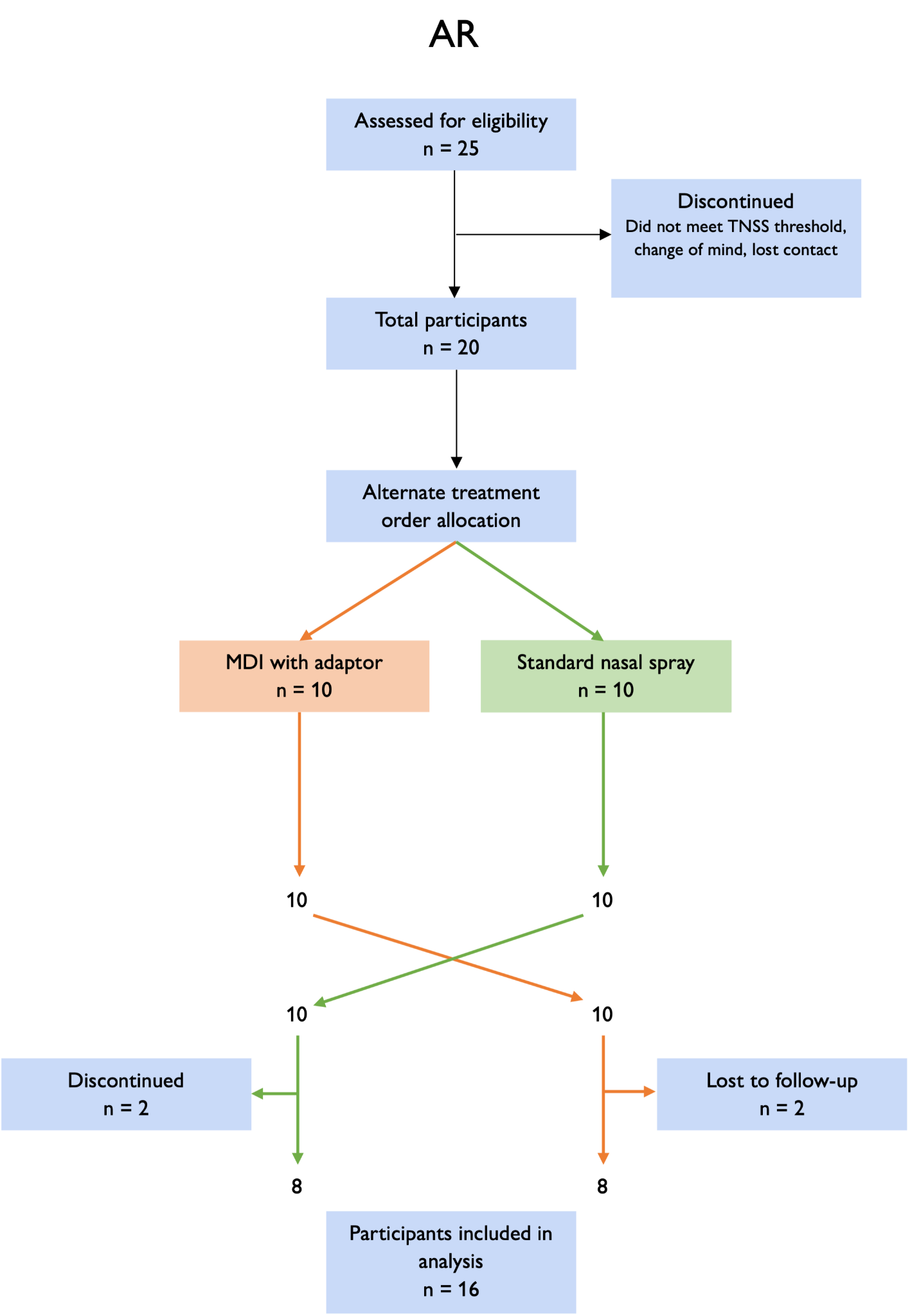

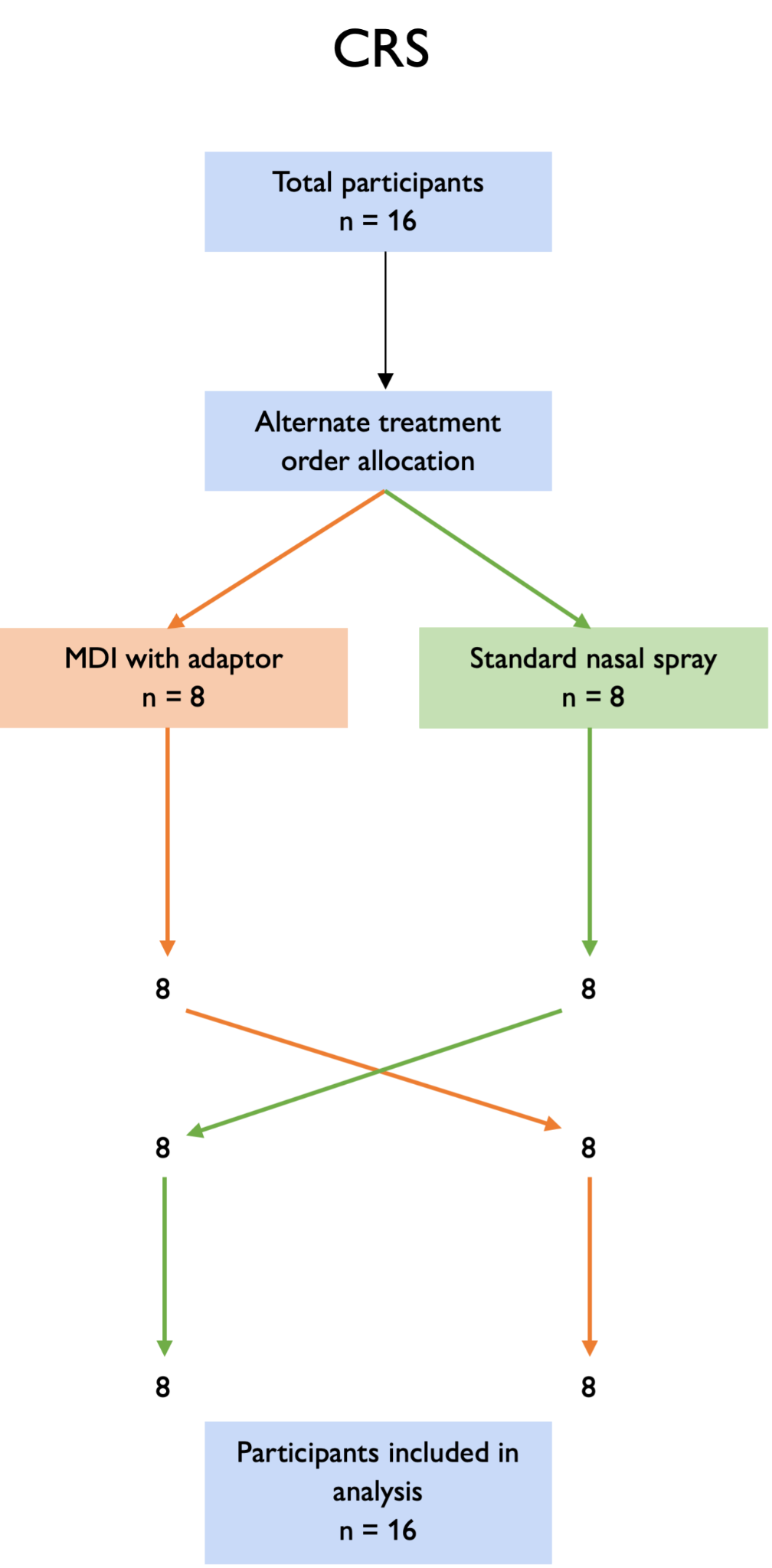


**Figure 3** Flowcharts demonstrating the study process in the CRS and AR trials. Participants were allocated to each device, then crossed over to the alternate device. A total of 16 participants completed the study in each trial.

# **SUPPLEMENTARY TABLES**

**Table 1** Participant characteristics in the CRS and AR trials

|  | **AR (n = 16)** | **CRS (n = 16)** |
| --- | --- | --- |
| **Female (n, %)** | **13 (81%)** | **12 (75%)** |
| **Baseline TNSS score (n, %)** | **4 - 5 : 1 (6.25%)**  **6 - 7 : 8 (50%)**  **8 - 9 : 3 (18.75%)**  **10 - 12 : 4 (25%)** |  |
| **Baseline SNOT-22 score (n, %)** |  | **0 - 20 : 6 (37.5%**  **20 - 40 : 5 (31.25%)**  **40 - 60 : 5 (31.25%)**  **60 - 110 : 0 (0%)** |
| **CRS diagnosis** |  | **CRSwNP : 4 (25%)**  **CRSsNP : 7 (43.75%) Polyposis : 4 (25%)**  **Deviated septum and atelectasis of uncinate : 1 (6.25%** |
| **Operation type** |  | **Complete FESS : 14 (87.5%)**  **Mini-FESS : 2 (12.5%)** |
